# Impact of Climate and Soil on Plant Dynamics and Ecosystem Stability in Argan Orchards

**DOI:** 10.3390/plants14050664

**Published:** 2025-02-21

**Authors:** Maryem Telmoudi, Chaima Afi, Naima Chabbi, Said Labbassi, Assma Oumasst, Mohamed Ouknin, Rachid Bouharroud, Abdelaziz Mimouni, Mimoun El Kaoua, Driss Hsissou, Abdelghani Tahiri, Naima Ait Aabd

**Affiliations:** 1 Regional Center of Agricultural Research of Agadir, National Institute of Agricultural Research (INRA), Avenue Ennasr, BP415 Rabat Principale, Rabat 10090, Morocco; 2 Laboratory of Agrobiotechnology and Bioengineering, Department of Biology, Faculty of Science and Technology, Cadi Ayyad University, Gueliz, Marrakesh 40000, Morocco; 3 Laboratory of Biotechnology and Valorization of Natural Resources (LBVRN), Faculty of Sciences, Ibnou Zohr University, Agadir 80000, Morocco

**Keywords:** argan orchards, biodiversity indicators, alpha diversity, beta diversity, climate change, conservation

## Abstract

Assessing the impact of natural disturbances on plant biodiversity is crucial amid biodiversity loss and climate change. Research highlights dynamic shifts driven by environmental factors, climate change, and human activity, emphasizing the need to maintain ecosystem stability for biodiversity conservation and sustainable development, particularly in arid and semi-arid regions. This study, conducted between 2021 and 2023, focused on the dynamics of plant communities in argan tree reserve areas. Six argan orchards were selected as study sites for detailed investigation. A total of 82 species belonging to 25 families were identified, with 23 families documented in 2021 and 21 families in 2023, including six endemic species to Morocco (*Frankenialaevis* subsp. *velutina*, *Ononisnatrix* subsp. *arganietorum*, *Rumex papilio*, *Andryala integrifolia* subsp. *cedretorum*, *Chiliadenushesperius*, and *Reseda difussa*). The majority of the plants present in the study area were annual and biennial herbaceous types, exhibiting minimal seasonal stability within the plant communities. However, how communities respond to the effects of fluctuating disturbances remains unclear. This study explores the indirect effects of natural disturbances on community metrics in argan orchards, assessing plant diversity, biomass, and density across different orchard types. It highlights the influence of climate, soil properties, and biotic interactions on plant community dynamics. We utilized alpha diversity indices (Shannon, Simpson, Pielou’s, and Margalef’s) and beta diversity indices (Jaccard and Sorenson Similarity) to examine these patterns. Seasonal changes were predominantly influenced by temperature and precipitation, while diverse soil types shaped by relief, climate, and water balance contributed to different ecological functions. The key findings indicated the highest plant diversity in “Tioughza” and the most significant plant density in “Imoulass” and “Ezzaouite”. Soil nutrients (N, C, and P) showed a positive correlation with plant biomass, highlighting their vital role in biomass accumulation, whereas temperature, C/N ratio, and loam percentage were found to be crucial for plant richness. Mixed modeling revealed a significant relation between density and biomass, but no significant effect between alpha diversity (Shannon Index) and elevation. This study concludes that soil texture and climate significantly shape the relationships between diversity, biomass, and density, recommending further research into interactions among plant diversity, cover, biomass, and soil fertility to support the sustainable management of argan orchards.

## 1. Introduction

Dryland forests, which account for 6% of the world’s forests, provide key ecosystem goods and services to more than a billion people living within the arid and semi-arid climatic zones [1]. In recognition of its substantial ecological value and local economic importance, the Argan Reserve (RBA) was designated as a UNESCO Biosphere Reserve in 1998 [2,3]. This reserve is a refuge for the unique and naturally occurring argan forests, known as arganeraie [4]. The argan forest extends along the Atlantic seaboard in the west central part of Morocco, from the mouth of the Tansift valley in the north, to the mouth of the Draa Valley in the South [3,4], across the Souss plain, the southern slope of the western High Atlas and the northern slope of the western Anti-Atlas. It covers an area of around 2,560,000 ha representing all of the Arganeraie Biosphere Reserve [3].

The argan tree (*Argania spinosa* (L.) Skeels) is an endemic plant and the only representative of the tropical Sapotaceae family in Morocco [4,5]. It is widely adaptive to arid and semi-arid environments [6] and prolonged drought [7]. The argan tree plays an important role in the biodiversity of the forest ecosystem [8]. It is capable of withstanding extremely high temperatures, reaching up to 50 °C in most continental regions [9,10]. However, it is also dependent on a certain quantity of water to survive and flourish [10]. It is primarily found in regions with low rainfall, with mean annual values ranging from100 mm [2,3] to 400 mm [3,9,10]. Morocco is a country with a Mediterranean climate where rainfall occurs during the period from September to April (cool season). There are two distinct seasons, a rainy season (September–April) and a dry season (more than 8 months) [9].

Generally, the ecosystem goods provided by dryland forests include fodder, fuelwood, medicines, herbs, and other forest products, as well as marketable goods. They also include soil stabilization, climate change mitigation, water conservation, and the fight against erosion and desertification [1]. Specifically, the argan woodlands perform a variety of ecosystem services, the most important being carbon storage and sequestration, conservation of genetic diversity, food and water, local hydrological processes, securing soil fertility, prevent soil erosion, regulation of microclimate, and habitat for species, and provide the final barrier against desertification [3]. The species composition of forest stands has been shown in numerous studies to be the main driver of forest biodiversity. It can directly determine the diversity and composition of the regeneration layer. Furthermore, understory vegetation is linked to tree species composition through complex pathways (light, fall, litter, etc.) [11]. Several authors have demonstrated the importance of argan forest products for local populations, particularly the rural population [1]. On the other hand, thanks to its geographical location and the influence of different climates, Morocco boasts a rich flora. Nearly 5200 species and subspecies of vascular plants have been recorded, grouped into 155 families and 981 genera. In terms of endemism, the number of endemic species in the flora is 900 species, and the Moroccan medicinal flora is estimated to have600 species [12].

However, climate change and biodiversity loss are major interconnected environmental challenges facing the world today. It has been proven that conservation measures can simultaneously significantly mitigate anthropogenic climate change [13]. The managers report that biological diversity in protected areas is threatened in 27% of cases [14]. Global warming and reduced water availability are major risk factors for tree loss and forest dieback. In the semi-arid region of Morocco, water scarcity is a major factor limiting productivity. More than two-thirds of Morocco can be classified as arid and semi-arid, with low and variable rainfall and frequent droughts. Prolonged drought has had adverse ecological consequences, including loss of vegetation, deforestation, and falling water tables in some regions [2]. Perhaps the most serious aspect of the environmental crisis is the loss of biodiversity. This loss disrupts essential ecosystem services such as crop pollination and water purification and destroys culturally important species [11]. According to De Waroux et al. [1], argan forests are experiencing high rates of change, with a spectacular drop in the density of more than 40% between 1970 and 2007. Additionally, climate change-induced tree mortality alters understory plant communities and soil biota, leading to a greater proportion of shaded species. Many of these species are shade-intolerant and have shorter lifespans, though the duration of these changes is not well understood [12]. The Argan Biosphere Reserve has been hit hard in recent years by long and intense heat waves, causing loss of vegetation cover and changes in land use resulting in erosion [9]. Forests are a refuge for the majority of terrestrial biodiversity, which is why preserving forest biota is crucial to global biodiversity conservation [11]. Several physical and anthropogenic factors are reducing the density and surface area of argan ecosystems, thus reducing the biodiversity of natural spaces [8]. A better understanding of the dynamics of land use change in these ecosystems seems necessary.

Local capacity to manage biodiversity and ecosystem services is lacking [14]. However, it is still possible to avoid dramatic biodiversity degradation and the subsequent loss of ecosystem services through intensified conservation efforts [15]. Indeed, large-scale investments in reforestation and “arganiculture” aim to conserve biodiversity and mitigate carbon emissions. Nevertheless, Morocco’s market-driven reforestation program has mainly benefited consumers and industry leaders, rather than local producers and their ecosystems. Additionally, the convergence of various interests in the argan forest, under the guise of combating degradation, has increased tensions among the goals of biodiversity conservation, economic production, and human development [16]. Hence, the project to develop the argan forest through “arganiculture: DARED” or “Development of Argan Orchards in Degraded Environment” was proposed by Morocco to enhance biodiversity conservation and mitigate carbon emissions through the planting of 25,000 acres of argan trees.

This research on plant biodiversity in the argan orchards planted in the bordering natural forest aimed to assess its current status by focusing on the impact of management systems in six argan tree orchards in vulnerable areas. Over two years with varying climatic conditions, differences in structural variations, soil properties, and plant composition were observed. After the introduction of argan trees aimed at revitalizing the ecosystem, a study was conducted on the biodiversity linked to this prominent species within its orchards. The timeframe for the recovery of forest ecosystems following a decline in biodiversity is still uncertain, as is our capacity to expedite this recovery process.

This study hypothesized that both macro-scale and micro-scale factors, including interspecific interactions and stand structures, influence biodiversity control. It integrated the assessment of biological richness and threats to ecosystems, analyzing biodiversity indices to understand management importance and conserve these ecosystems. Six platforms were chosen under similar conditions to reduce confounding factors, although pre-existing differences were acknowledged. By examining pedoclimatic factors and biodiversity indices, this study sought to prevent biodiversity degradation and loss of ecosystem services. A fine-scale analysis of environmental conditions and plant species distribution aimed to support effective forest management policies.

To fill these gaps, the present study aimed to examine the relationships between plant biodiversity, climate, and soil to achieve a better understanding of the existing conditions and provide answers to these important questions. In what ways do management practices and climate in argan orchards affect plant species diversity, structural diversity, diversity indices, and soil characteristics over a two-year period? What valuable insights can be derived from these results to improve future management and conservation approaches for *Argania spinosa* ecosystems?

## 2. Materials and Methods

### 2.1. Study Site

This study was conducted in six separate argan orchards across the marginal and vulnerable areas within RBA forests in three Moroccan regions: Guelmim-Oued-Noun, Souss-Massa, and Marrakech-Safi (Table 1). These regions span semi-arid, arid, and pre-arid (Saharan) climate zones, with altitudes ranging from 180 to 1207 m (Table 1). The average temperature is 18.5 °C, with monthly variations between 9.4 °C and 26 °C [6,9]. Annual precipitation averages 301.6 mm, mostly occurring from October to April [9]. Details of their geographical location, elevation, and soil composition are presented in Table 1.

The study area features diverse topography, with significant variations across regions (Table 1). From rolling hills and valleys to rugged mountains and plains, the landscape is highly heterogeneous. This topographical diversity not only enhances the area’s beauty but also influences its ecology, hydrology, and local livelihoods, making it a valuable subject for further study.

### 2.2. Experimental Design

**Figure 1:**
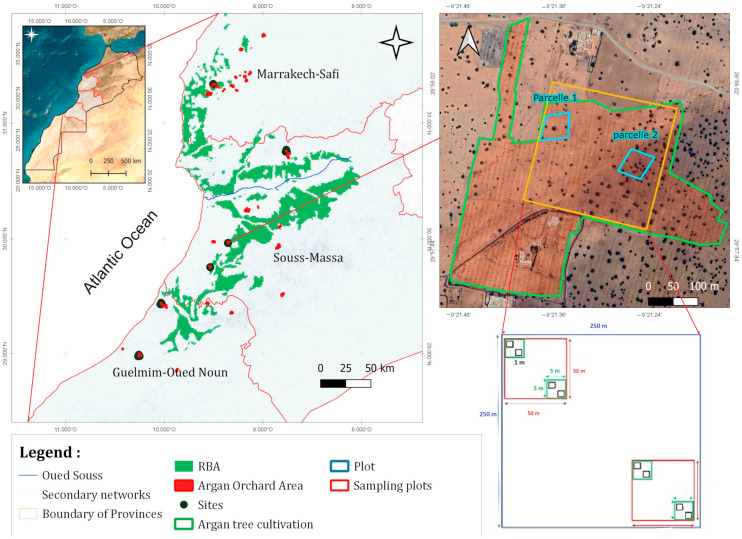
Geographic location of the study area in Central-Western Morocco.

The same methodology was applied across the six argan orchards (Figure 1) using the Gentry plot method [17], with minor modifications. At each site, a 250 m × 250 m plot (6.25 ha) was established. Within this plot, two 50 m × 50 m sampling units were positioned diagonally opposite each other. In each sampling unit, two 5 m × 5 m sub-plots were delineated, and within each sub-plot, two-fixed 1 m × 1 m quadrats were used for inventory (Figure 1), resulting in a total of 48 quadrats.

The sites were established in orchards planted with argan trees in 2020, and all plots were precisely located using GPS coordinates. Fixed quadrats measuring 5 m × 5 m and 1 m × 1 m were set up, with the 1 m × 1 m quadrat designated for inventory purposes throughout all years of monitoring. This sampling design ensures a comprehensive assessment of the area’s diversity and richness.

Additionally, three 1 m × 1 m quadrats were randomly selected within each site for biomass (fresh weight) data collection. Data were collected in March 2021 and March 2023.

### 2.3. Plant Diversity and Vegetation Analysis

All herbaceous plants within the quadrats were recorded, collected, and taxonomically identified by their scientific names. Most species were identified directly in the field during the vegetation survey. For species that could not be reliably identified on-site, voucher specimens were collected. Leaf samples and reproductive organs of unidentified species were gathered, pressed, and dried. The samples were placed in labeled cardboard bags in the field and subsequently transported to the INRA laboratory for further identification, using appropriate reference materials.

### 2.4. Stand Structural Indices: Diversity Analysis

In each study site, community diversity was assessed for herb species only, at the quadrat level (1 m × 1 m). Diversity indices were then calculated at the plot scale (5 m × 5 m) based on four replicates.

#### 2.4.1. α-Diversity

The alpha diversity indices, including species richness (S), which represents the algebraic count of species (i) per sample, Shannon–Wiener diversity index (H), Pielou’s evenness index (J), Simpson’s dominance index (D), and Margalef’s richness index (R), were calculated.

The Shannon diversity index (H′) is a measure of biodiversity that is sensitive to rare species [18]. It was determined by the following formula:

$$H^{\prime }=\sum_{i=1}^{s}PiLnPi$$

where Pi is the proportion of species (i) in the sample, and ranges such as:

0 < H′ ≤ Ln(S)

The Margalef’s Index (R) was adopted and determined by the following formula:

R=(S−1)/Ln(N)

where R is the richness, N is the total number of individuals in a community of a certain plot, and S is the total number of species. The classification of richness is as follows: R > 2: high richness; 1< R < 2: medium richness; R < 1: low richness.

The Simpson index [19] is a measure of biodiversity that is sensitive to abundant species, and varies from 0 ≤ D < S. It was determined by the following formula:

$$D=1-\sum_{i=1}^{s}Pi²$$

Pielou evenness (J): resulting in a value constrained between (0 and 1). The less variation in communities between the species, the higher J is. In other words, an even community is 1.

$$J=H^{\prime }max/\ln(S)$$

#### 2.4.2. β-Diversity

β-diversity refers to the diversity between different ecological communities. In terms of β-diversity patterns, the Jaccard dissimilarity index (βjac) was used to examine differences between all sites and species that were quantified based on species presence–absence data. β-diversity was also assessed using the Sorensen dissimilarity index (βsor).

#### 2.4.3. Structural Diversity Analysis

The vegetation structure was assessed at the plot scale of 250 m × 250 m, with two or three 1 m × 1 m quadrats randomly selected for calculated biomass collection. The fresh weight (g/m^2^) (FW) data for the understory, plants, and fine roots were recorded. Two quadrats were employed to calculate density (plants per 2 m^2^), and four replicates were conducted at each site.

### 2.5. Soil Sampling and Analyses

For soil sampling, four random locations were chosen within each 250 m × 250 m plot on six platforms. Soil samples were collected in March 2021 and 2023 at depths of 0–5, 5–10, and 10–30 cm using a standard soil auger. The samples were placed in plastic bags, labeled in the field, and sent to the INRA soil laboratory for analysis 15 days later. The main parameters analyzed included organic matter (OM), organic carbon (OC), total nitrogen (N), available phosphorus (Pava), electrical conductivity (EC), and pH. The Carbon/Nitrogen ratios (C/N), which were calculated and used as indicators of soil fertility. Soil texture (sand, silt, and clay content) was also assessed once in 2021 for site classification.

### 2.6. Climate Data Analysis

The climate data were collected for the cool seasons (November to March) of 2020–2021 and 2022–2023 for all studied sites. The climate data were extracted from the NASA Global Climate Data Platform (https://power.larc.nasa.gov/data-access-viewer/, accessed on 15 February 2024), for all parameters except the precipitation we use the data platforms (https://www.historique-meteo.net/, accessed on 6 April 2024) (Figure S1). The parameters for the rainy season were assessed, including temperature range, mean, minimum, and maximum values (°C), specific humidity (g/kg), relative humidity (%), wind speed (mean, maximum, and minimum, in km/h), and monthly precipitation (mm). Additionally, the total seasonal precipitation (mm) was calculated (Figure S1).

### 2.7. Data Analyses

The research was conducted in two distinct phases. The first phase involved fieldwork to identify the characteristics of herbaceous species in each quadrat. The second phase focused on processing the collected data-using version 4.3.1 in R software.

Pairwise comparisons: A Student’s *t*-test was used to compare means and assess the effect of years (inter-group factor) on each site independently, with Shapiro–Wilk and Levene’s tests applied for data validation. A general linear model (GLM) analyzed the intra-group effects of sites (fixed factor) on various dependent variables, including environmental factors and diversity indices for the two years independently. Duncan’s multiple-range posthoc tests verified the significance of mean differences using the “agricolae” package version ‘1.3.7’.

To account for the hierarchical structure of the sampling design, sites were included as random intercepts in the linear mixed models (LMMs). Model performance was evaluated using a simulation-based approach to residual diagnostics, assessing randomized quantile residuals for linearity, uniformity, and homoscedasticity through diagnostic plots. To meet model assumptions, data on density, fresh weight (FW), and elevation (Elv) from the most severely disturbed sites were log-transformed. Additionally, we tested for multicollinearity and confirmed that all variance inflation factors (VIFs) were below 2.2, indicating stable model coefficient estimates. Model parameters were estimated using restricted maximum likelihood (REML), and the statistical significance of the LMMs was determined using F-tests with Satterthwaite’s approximation for degrees of freedom. We used the ‘lme4’ package version ‘1.1.34’ [20].

Principal component analysis (PCA) was used to explore relationships between all ecosystem variables, conducted with the “FactoMineR” and “factoextra” packages. Spearman correlation assessed relationships among soil elements, plant density, fresh weight (FW), diversity, and climate.

The hypothesis that species composition varies by site was tested using non-metric multidimensional scaling (NMDS) from the ‘vegan’ package, employing Bray–Curtis distance for dissimilarity calculations. The resulting ordination diagrams were presented in two dimensions (k = 2), and the model fit was assessed using stress values. To assess the significance of compositional differences, permutational multivariate analysis of variance (PERMANOVA) was employed. In each year, sites were treated as a factor in the PERMANOVA analysis. Additionally, a permutational analysis of multivariate dispersions (PERMDISP) was conducted to evaluate if group dispersion influenced the significance of compositional differences. NMDS, PERMANOVA, ANOSIM, and PERMDISP (PERMutationalDISPersion) analyses were executed using the metaMDS(), vegdist(), adonis(), and betadisper() functions, respectively, within the ‘vegan’ package version ‘2.6.4’ [21]. All statistical analyses were carried out under the R environment, version 4.3.1 in R.

## 3. Results

### 3.1. Comparison of Soil Physiochemical Properties in Different Orchards

#### 3.1.1. Comparison of Soil Physiochemical Properties in Different Sites for Each Year: Effect of Site

**Figure 2:**
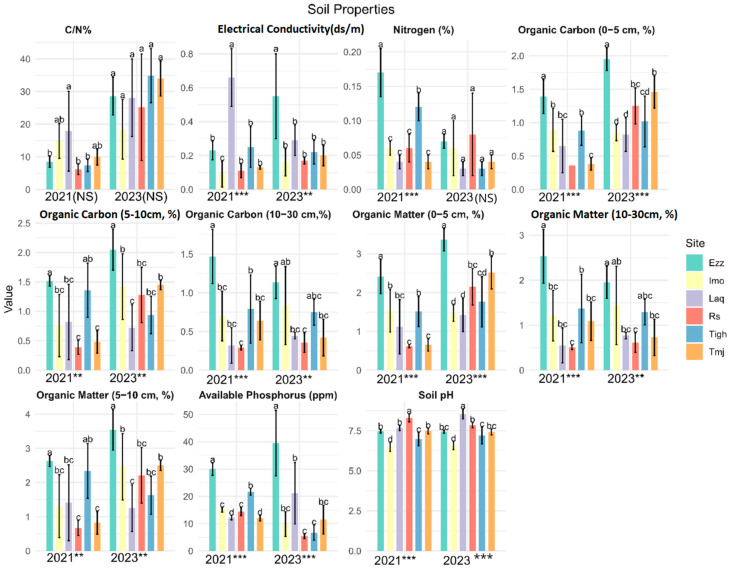
Mean values (±standard error) soil properties of the six-orchards for each twoyears 2021 and 2023 (*** significant at *p* < 0.001; ** significant at *p* < 0.01, NS: no significant *p* > 0.05). (a, b, c, d is results of test post hoc Duncan). Rs: Rasmouka, Tmj: Tamjloujt, Tigh: Tioughza, Laq: Laqsabi, Imo: Imoulass, Ezz: Ezzaouite, C/N:Carbon–Nitrogen ratio, N: Nitrogen, OM: organic matter, OC: organic carbon, EC: electrical conductivity, Pava: available phosphorus.

The GLM analysis of the physical and chemical soil matrices revealed notable variations between sites within the same years (Figure 2). Concerning soil properties across the six platforms, it was observed that the mean values of OM (0–5 cm), OC (0–5 cm), Pava, and PH were statistically significant differences between all the sites (*p*-value < 0.001) in two years.

The total nitrogen (Ntot) did not show significant differences between sites in 2023. Additionally, EC and OM-OC (10–30 cm) were statistically significantly higher across all sites in 2021 and showed moderate significance in 2023. Notably, the N/C ratio did not exhibit significant differences, while OM-OC (5–10 cm) demonstrated moderate significance between sites in both years.

#### 3.1.2. Comparison of Soil Physiochemical Properties in Different Orchards in Each Site: Effect Year

Test-T revealed a significant difference between all the measured variables of the soil properties except for OM and OC (10–30) (*p* > 0.05) (Table 2). However, this difference does not appear to be significant, with a minimal increase in Rs, Laq, and Imo, and a diminution in Tmj and Ezz. The Imo site demonstrated no discernible change in soil variables between 2021 and 2023. However, at the Laq site, changes were observed in pH and electrical conductivity, with an increase in pH and a decrease in electrical conductivity. Furthermore, the assimilable phosphorus and total nitrogen showed a notable decline, with augmentation of the C/N in Tigh, accompanied by a higher difference in significance < 0.001.

For soil properties, mean values of MO, OC (0–5 and 5–10 cm depth) and C/N ratio were significantly higher in Tmj, the same for OM, OC (0–5), and Pava in RS, but it is less significant for OM, OC (5–10), and pH. Considering soil properties, the mean values of OM, OC (at depths of 0–5 and 5–10 cm), and the C/N ratio were found to be significantly higher in Tmj. A comparable pattern was observed for OM, OC (0–5), and Pava in RS. Although the differences were less pronounced in Rs for OM, OC (5–10), and pH, the ratio C/N was not found to be significant.

### 3.2. Comparison of Structural Features

**Figure 3:**
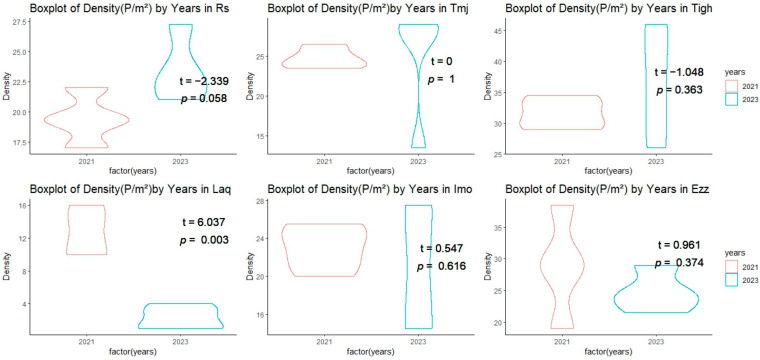
Comparison of the mean (±SE) density (P/m^2^) across sites between 2021 and 2023.

**Figure 4:**
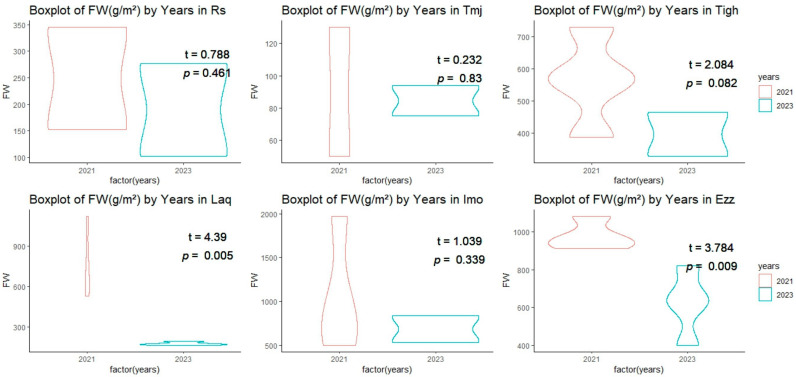
Comparison of the mean (±SE) the fresh weight (FW) (g/m^2^) across orchards between 2021 and 2023.

**Figure 5:**
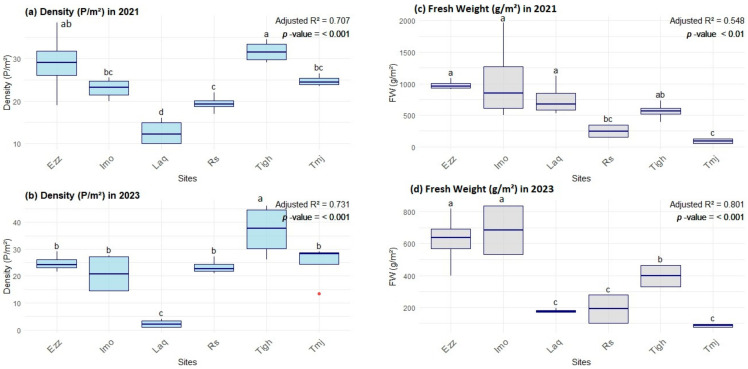
Means (±SE) of structural features: “Density” (plant/m^2^) and “Fresh weight” (FW) (g/m^2^) according to sites (Factor) in each of the two years 2021 and 2023, with adjusted R-squared and *p*-value. (**a**) Density in 2021, (**b**) Fresh weight in 2021, (**c**) Density in 2023 and (**d**) Fresh weight in 2023. Different lowercase letters indicate group differences between sites. Rs: Rasmouka, Tmj: Tamjloujt, Tigh: Tioughza, Laq: Laqsabi, Imo: Imoulass, Ezz: Ezzaouite.

Measurements of plant density and fresh weight (FW) at the six sites studied in 2021 and 2023 show significant variations (Figure 3, Figure 4 and Figure 5). Plant density values ranged from 2.25 plants/m^2^ at Laq in 2023 to 36.88 plants/m^2^ at Tigh in 2023. More specifically, Laq experienced a drastic decrease in density from 12.63 plants/m^2^ in 2021 to 2.25 plants/m^2^ in 2023, while Tigh saw an increase from 31.63 plants/m^2^ in 2021 to 36.88 plants/m^2^ in 2023. At Ezz, density remained relatively stable, with 28.88 plants/m^2^ in 2021 and 24.75 plants/m^2^ in 2023.

As for fresh weight (FW) measurements, similar trends were observed. At Imo, fresh weight dropped, from 1040 g/m^2^ in 2021 to 685 g/m^2^ in 2023. At Ezz, fresh weight fell from 978.86 g/m^2^ to 623.33 g/m^2^ over the same period. At some sites, such as Tigh, fresh weight also decreased, from 563.32 g/m^2^ in 2021 to 396.5 g/m^2^ in 2023. However, at Laq, despite a low plant density in 2023, fresh weight was measured at 177.33 g/m^2^, a significant decrease from 752.57 g/m^2^ in 2021.

#### 3.2.1. Inter-Group Comparisons of Structural Features at Each Site

A comparison of the means (test T) of the density matrix revealed significant differences between the two years in Laq, with a *p*-value of 0.003 and a t-value of 6.037 (Figure 3). This indicates a significant decrease in density in 2023 compared to 2021. The density is not influenced by the years in Rs, Tmj, Tigh, Imo, and Ezz, with a *p* value = 0.058 in Rs. The T value is negative in Rs and Tigh, indicating that the difference between the two years is negative and that the density in Rs and Tigh increased in 2023, although this is not statistically significant. In Tmj, Imo, and Tigh, we observed a dispersion of values in 2023 in comparison with 2021. However, no significant difference was noted. Nevertheless, we can explain this difference between the quadrats in the sites.

In terms of fresh weight, the results of the T-test for the Rs, Tmj, and Imo sites indicate that there is no significant difference between the two years, with a *p*-value greater than 0.05 and a T-value less than 1 (Figure 4). The same is true for the Tigh site, with a *p*-value of 0.08 and a T-value of 2.084. TheLaq and Ezz sites (T = 4.39 and 3.784, respectively) demonstrate a highly significant difference (*p* < 0.01) between the two years 2021 and 2023.

#### 3.2.2. Intra-Group Comparisons of Structural Features in Each Year

The analysis of the structural features (Figure 5) indicates that the mean density values for 2021 at the six sites are statistically significant, while the mean of the fresh weight is only marginally significant. Similarly, the mean fresh weight values for 2023 are statistically significant, while the mean density values are only slightly significant. It can be observed that when the density is found to be highly significant, the fresh weight becomes less significant, and vice versa.

#### 3.2.3. Effect of Density on Fresh Weight in Plant Density Is Strongly Associated

Surprisingly, LMM models reveal a significant effect of density on the fresh weight. An increase in plant density is strongly associated with an increase in fresh weight (FW), as indicated by the positive coefficient for density (t = 3.100, *p* = 0.003) (Table 3). This suggests that higher plant densities contribute to greater fresh biomass production. Conversely, the year 2023 is associated with a significant decline in fresh weight compared to 2021, as evidenced by the negative coefficient for years (t = −3.282, *p* = 0.002). This decline implies that environmental and ecological conditions in 2023 had an adverse effect on biomass accumulation. Additionally, the variance associated with the sites (0.7873) highlights significant differences in fresh weight among locations, suggesting that certain sites support higher biomass production than others, independent of the effects of plant density and year. These findings emphasize the role of both density-dependent growth patterns and external environmental factors in determining fresh biomass yield.

### 3.3. Comparison of Alpha Diversity Indices in Different Sites for Each Year

**Figure 6:**
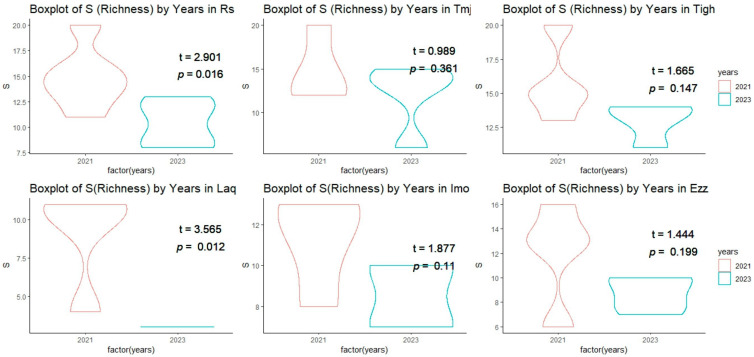
T-test comparison of the mean (±SE) richness index across sites between 2021 and 2023.

**Figure 7:**
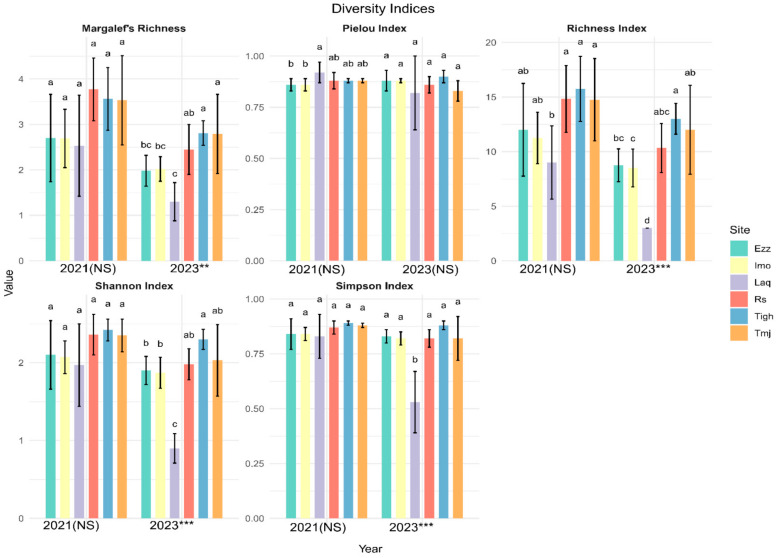
Mean values (±standard error) of the six sites diversity index in each year 2021 and 2023 (*** significant at *p* < 0.001; ** significant at *p* < 0.01; NS: not significant *p* > 0.05). (a, b, c, d is results of test post hoc Duncan). Rs: Rasmouka, Tmj: Tamjloujt, Tigh: Tioughza, Laq: Laqsabi, Imo: Imoulass, Ezz: Ezzaouite.

Plant diversity indices were assessed at six sites in 2021 and 2023 (Figure 6 and Figure 7, Table 4). The indices measured include species richness (S), Shannon index, Simpson index, Pielou index, and Margalef’s richness index. In 2021, species richness was highest at Tigh (15.75 ± 2.98), followed by Rs (14.83 ± 3.06) and Tmj (14.75 ± 3.77). At Laq, richness was lowest, with a score of 9.00 ± 3.36. In 2023, a significant decrease in richness was observed at several sites, including Laq, where it fell to 3.00 ± 0.00. At Tigh, richness remained relatively high at 13.00 ± 1.41.

#### 3.3.1. Inter-Group Comparisons of Alpha Diversity at Each Site

A comparison of the averages from the student test conducted at the same site across different years revealed a significant difference (*p* < 0.05) (Figure 6) in the index of richness between the sites of Rs and Laq, with a T value of 2.90 and 3.56, respectively. However, no significant difference was observed in the sites of Tmj, Tigh, Imo, and Ezz (Figure 6).

Table 4 shows that the change in diversity is significantly different only at the Rs and Laq sites, with notable discrepancies observed in the Shannon, Simpson, and Margalef’s indices. The Margalef’s index is highly significant at the Rs site, while the Shannon index is highly significant at the Laq site. However, Pilou’s equitability index does not show any significant differences across all sites. Additionally, there is no variation in the diversity index for the Tmj, Tigh, Imo, and Ezz sites.

#### 3.3.2. Intra-Group Comparisons of Alpha Diversity in Each Year

The diversity index results for 2021 indicate that there are no significant differences among all sites, as the *p*-values for all indices exceed 0.05. Specifically, the richness has a *p*-value of 0.058, Margalef’s index is at 0.144, the Pielou index is 0.175, the Shannon index is 0.256, and the Simpson index is 0.530 (Figure 7). However, in 2023, the *p*-values for the Simpson Index, Shannon Index, S Index, and Margalef’s richness are all below 0.05, indicating significant differences between the sites for these indices. The *p*-value for the Pielou Index is slightly above 0.05, suggesting a marginal difference that may warrant further investigation.

#### 3.3.3. Effect of Elevation in Alpha Diversity (Shannon Index)

Linear mixed models (LMM) were performed to analyze the relationship between elevation and the Shannon Index. According to our results, elevation did not have a significant effect on the Shannon index. This may be justified by the absence of a wide altitudinal range in our data or by the low impact of altitude variations on the variable measured during those years. Alpha diversity was compared between the two years classes in different elevation on the basis of H′.

Conversely, the study sites appeared to exert no negligible effect, as indicated by the values of variance (0.1133) (Table 5). The years have a significant effect in alpha diversity (t value = −4.347, *p* < 0.001). The altitude and years explains 15% of variance (R^2^marginal = 0.150), and with random effect the model explains 60% (R^2^conditional = 0.601). This diversity is influenced not only by the altitude, but also by the specific characteristics of individual study sites, which acted as random factors. In other words, the variations in diversity H′ is influenced by multiple factors and unique site-specific characteristics. Another possible cause could be the soil type.

### 3.4. Comparison of Climatic Conditions in Analyzed Orchards

**Figure 8:**
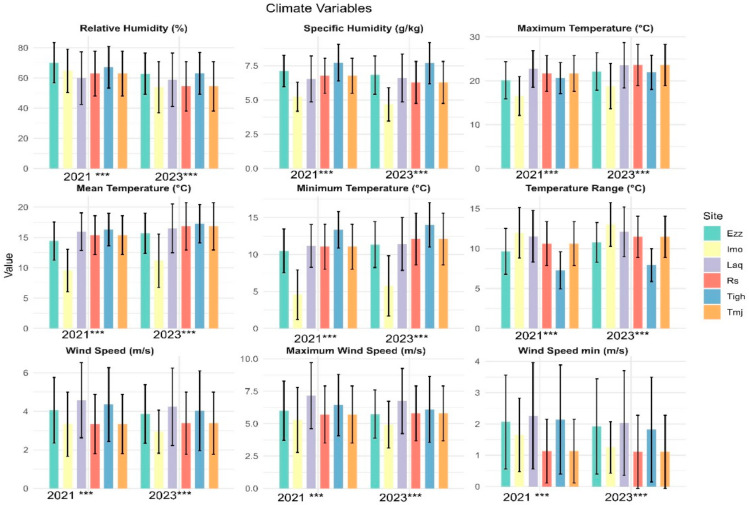
Mean values (±standard error) climate parameter of the six sites in each two years 2021 and 2023. (*** significant at *p* < 0.001). Rs: Rasmouka, Tmj: Tamjloujt, Tigh: Tioughza, Laq: Laqsabi, Imo: Imoulass, Ezz: Ezzaouite.

The climate parameter results for the two years indicate a statistically significant difference across all sites for all parameters (Figure 8), except for Pp (Table 6) in the same years, but 2021 was rainier than 2023 (Figure S1). The temperature ranges are as follows: 7.27 °C in Tigh, 9.65 °C in Ezz, 10.62 °C in Rs-Tmj, 11.53 °C in Laq, and 11.95 °C in Imo, with a statistically significant increase observed in 2023. Laq has an average maximum temperature (Tmax) of 22.7 ± 4.18 °C, while Imo has an average minimum temperature (Tmin) of 4.57 ± 3.35 °C.

### 3.5. Relationships Between the Index Diversity Relationship with Ecological and Environmental Factors

#### 3.5.1. Principal Component Analysis

**Figure 9:**
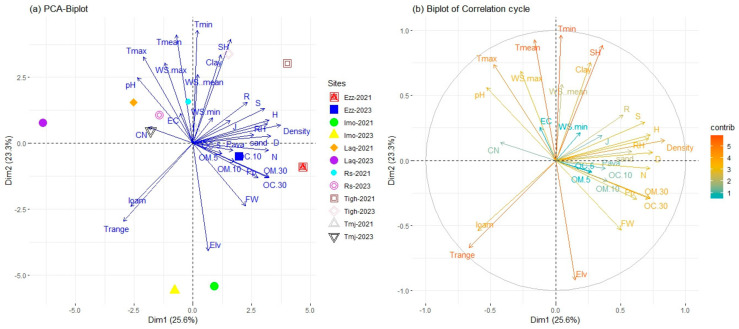
Biplot of principal component analysis (PCA) applied the difference site in two years; Richness (S), Shannon diversity index (H′), Simpson diversity index (D) Pielou diversity index (J), Margalef’s richness (R), Plant Density (Density), Fresh weight (FW), available phosphorus (Pava), total nitrogen (N), PH, Electrical conductivity (EC), organic matter in three depth OM (0–5), OM (5–10), OM (10–30), organic carbon in three depth OC (0–5), OC (5–10), OC (10–30), carbon/nitrogen (C/N), Elevation (Elv). And variable climate: T range, Tmean, minimum and maximum temperatures (Trange, Tmean, Tmin, and Tmax), respectively, specific humidity (SH), relative humidity (RH), wind speed (WS) (WS mean, WS max, and WS min), and precipitation mean (Pp). As well as the soil texture: sandy, clay, and silt percentage. (**a**) PCA Biplot, (**b**) Biplot of Correlation cycle.

The distribution of the plots according to the two years is displayed on the factorial map of the PCA (Figure 9). The first and second axes accounted for 48.9% of the explained variance (25.6% and 23.3%, respectively).

The multivariate analysis results revealed significant correlations between various environmental and soil parameters and the principal components. Density, Simpson diversity index (D), Shannon diversity index (H), organic matter (10–30 cm), organic carbon (10–30 cm), relative humidity (RH), richness (S), temperature range (T range), and mean precipitation (Pp) were strongly associated with the first axis (PC1). Climate parameters such as minimum and mean temperature (Tmin and Tmean), elevation, specific humidity (SH), clay content, maximum temperature (Tmax), maximum wind speed (Wsmax), temperature range (T range), mean wind speed (WSmean), and pH were primarily correlated with the second axis. Sites such as Ezzaouite (Ezz) and Tighin in both 2021 and 2023, along with Imoulass (Imo) in 2021, were positioned on the positive side of PC1 (Figure 9a). These sites exhibited higher values for density (0.84), Simpson diversity index (0.74), Shannon diversity index (0.73), organic matter (10–30 cm) (0.73), nitrogen (N), organic carbon (10–30 cm) (0.72), relative humidity (0.72), richness (0.68), and precipitation (0.62), which were the main variables associated with this group. Conversely, sites such as Laq in both years (2021 and 2023), Tmj in 2021, Rs, and Imo in 2023 were positioned on the negative side of PC1. This group was characterized by higher values for temperature range (−0.66), loam content (−0.60), and pH (−0.53) (Figure 9a,b).

The PC2, which is on the positive side, is characterized by the variables with the highest contribution, namely Tmin (0.96), Tmean (0.93), SH (0.88), clay (0.75), and Tmax (0.73). The WSmax, WSmean, and pH with values of 0.69, 0.58, and 0.56, respectively, are also noteworthy. The site of Laq in 2023 is distinguished by an elevated ratio of carbon to nitrogen C/N, pH, and lower rainfall. Furthermore, on the negative side of the PC2, the plots demonstrate that the Imo is separated from the other sites with a higher elevation (Table 1) and fresh weight, as indicated by Elv (−0.92), Trang (−0.67), and FW (−0.53) (Figure 5 and Figure 9).

FW demonstrated a positive and similar correlation with organic matter and organic carbon across all depths, with a particularly strong association observed at the 10 to 30 cm depth. This depth and biomass also exhibited a significant correlation with elevation. No correlation was observed between fresh weight and specific humidity or clay percentage. However, pH, EC, and Tmax exhibited a positive correlation between them, while fresh weight demonstrated a negative correlation with pH. A total nitrogen exhibit is inversely correlated with the C/N ratio and positively correlated with Pava. Elevation is negatively correlated with temperature (max, mean, and min) and wind speed. However, it is positively correlated with loam and temperature range. In addition, the latter is inversely correlated with specific humidity. The correlation between clay and loam is negative (Figure 9).

The diversity indices, OM-OC, and all other variables, including N, Pava, density, biomass, sand, relative humidity, and precipitation, exhibit positive correlations. A significant proportion of the total contribution is attributed to Elv, T(max, mean, rang, min), specific humidity, density, S, H, D, Pp, OM-OC 30 cm, relative humidity, biomass, and N. Organic matter and organic carbon at a depth of 10–30 cm show a strong correlation with rainfall.

#### 3.5.2. Pearson’s Correlation

**Figure 10:**
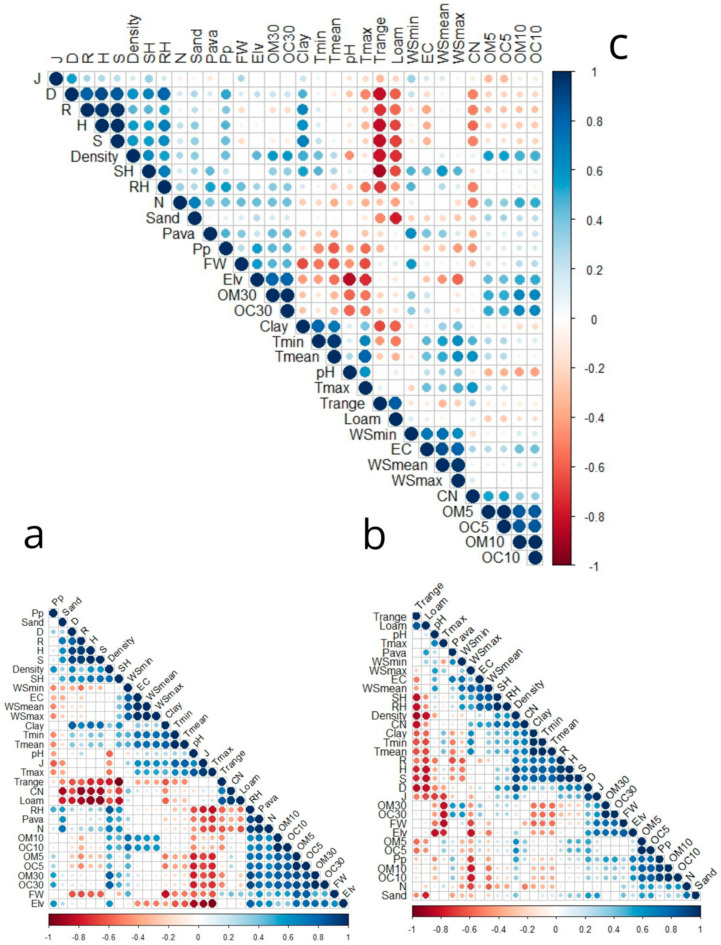
Map of the Spearman correlations among the environmental, Richness (S), Shannon diversity index (H′), Simpson diversity index (D) Pielou diversity index(J), Margalef’s richness (R), Plant Density (Density), Fresh weight (FW), available phosphorus (Pava), total nitrogen (N), PH, Electrical conductivity (EC), organic matter in three depthOM (0–5), OM (5–10), OM (10–30), organic carbon in three depth OC (0–5), OC (5–10), OC (10–30), carbon/nitrogen (C/N), Elevation (Elv). And variable climate: T range, Tmean, minimum and maximum temperatures (T range, Tmean, Tmin, and Tmax), respectively, specific humidity (SH), relative humidity (RH), wind speed (WS) (WS mean, WS max, and WS min), and precipitation mean (Pp). As well as the soil texture: sandy, clay, and silt percentage. (**a**) In 2021, (**b**) In 2023, (**c**) In two years.

The results of Pearson’s correlation (Figure 10) show that pH (Figure 10c) exhibits a positive correlation with maximum temperatures (Tmax) and mean temperatures (Tmean), while displaying a negative correlation with fresh weight (FW) and elevation.

Organic matter (OM) and organic carbon (OC) (Figure 10c) exhibit a strong correlation across all depths, highlighting their interconnected role in soil biological processes. The C/N ratio (Figure 10c) shows a negative correlation with the Margalef index, Shannon index, Richness index, relative humidity (RH), nitrogen, and precipitation (Pp), suggesting that these factors collectively influence nutrient cycling. In contrast, the C/N ratio is positively correlated with Tmax and OM-OC 5, indicating that temperature and organic carbon dynamics significantly affect soil nutrient efficiency. Electrical conductivity (EC) (Figure 10c) is positively correlated with wind speed (minimum, average, and maximum), suggesting an interaction between air movement and soil ion concentration. Additionally, EC shows positive associations with Pava and the Evenness index (J), reflecting its influence on soil structure and nutrient distribution.

The parameter “Pava” (Figure 10c) correlates positively with EC, OM, and OC at all depths (5, 10, and 30 cm), as well as with minimum RH, wind speed, and total nitrogen, underscoring its role in enhancing soil organic structure. Total nitrogen (Figure 10c) is positively associated with sand, RH, OM, and OC, particularly at depths of 10–30 cm, but shows a negative correlation with the C/N ratio and loam content. Conversely, sand (Figure 10c) is negatively correlated with loam but positively associated with soil density and total nitrogen, emphasizing its role in influencing soil texture and nutrient availability.

Tmax (Figure 10c) shows a negative correlation with rainfall (Pp), fresh weight (FW), organic matter (30), and elevation (Elv), while positively correlating with Tmin, Tmean, WSmax, C/N, and pH, indicating a relationship between maximum temperatures and thermal variations in the soil. The temperature range (T range) (Figure 10c) exhibits a positive correlation with the percentage of silt, but is negatively correlated with clay, density, specific and relative humidity, and the diversity index (S, H, D, R). This indicates that daily temperature variations directly influence soil texture and biodiversity. Silt is also negatively correlated with clay, sand, density, nitrogen, T (min, mean), and specific humidity, adversely affecting Shannon richness, dominance index, R, and H.

Tmin (Figure 10c) presents a negative correlation with elevation, biomass, silt, and temperature range (T range), but is positively correlated with temperature (mean–max)clay, mean-maximum wind, and specific humidity. This demonstrates that minimum temperatures are influenced by both soil texture and atmospheric conditions. The average temperature (T mean) shown in Figure 10c exhibits a positive correlation with pH, minimum and maximum temperatures, maximum mean wind speed, clay content, and specific humidity. In contrast, it demonstrates a negative correlation with elevation, rainfall, temperature range, loam, and fresh weight (FW), suggesting that the average temperature is significantly affected by the soil’s physical and chemical properties.

The maximum wind (Figure 10c) shows a positive correlation with wind speeds (minimum and mean), electrical conductivity (EC), pH temperatures (minimum, maximum, and average), and specific humidity, while it displays a negative correlation with elevation. Regarding specific humidity (Figure 10c), it is positively related to wind (maximum, minimum, and average), Tmin, and Tmean, along with clay, but negatively correlated with temperature range and silt.

A positive correlation was observed between relative humidity (Figure 10c) and precipitation, specific humidity, biomass, diversity index (R and S), total nitrogen, Pava, and OM-OC 30 in the soil, but it exhibited a negative correlation with temperature range and ratio C/N, suggesting that humid conditions favor nitrogen accumulation. The amount of precipitation (Pp) has a positive correlation with all diversity indices, except for Pielou’s evenness, which does not show a correlation with Pp. Additionally, precipitation is positively associated with nitrogen, OM-OC30, and relative humidity, while it is negatively associated with the Tmean-max C/N ratio, highlighting the effect of precipitation on soil chemical composition and biodiversity.

Elevation (Figure 10c) demonstrates a negative correlation with pH, clay, temperatures (maximum, minimum, and average), as well as maximum-mean wind speed, indicating that higher altitudes are generally associated with cooler and less acidic conditions. However, elevation is significantly positively correlated with fresh weight (FW), suggesting that plants at higher altitudes retain more moisture, possibly due to lower temperatures.

Density (Figure 10c) shows a positive correlation with various diversity indices such as dominance, Marglaf’s richness, species richness, and the Shannon index, indicating the plant’s density promotes greater biological diversity. The density is also positively correlated with OM-OC30, reinforcing the idea that organic matter and density positively influence biodiversity. It is also slightly correlated with sand and clay.

Conversely, temperature range, pH, and loam percentage are negatively correlated with density, indicating that lower plant density is associated with silt-textured soils and greater temperature fluctuations.

Fresh weight (Figure 10c) shows a positive correlation with elevation, indicating that plants located at higher altitudes tend to hold more water. Conversely, it has a negative correlation with rising mean and maximum temperatures, alongside pH levels, implying that increased temperatures and more acidic soils hinder plants’ capacity to maintain moisture.

The J regularity index (Figure 10c) is positively correlated with species richness, electrical conductivity, and dominance index, indicating that a more balanced distribution of species is associated with a higher Simpson index. Conversely, a lower Simpson index reflects a more pronounced dominance of certain species. The dominance index itself shows a positive correlation with all diversity indices (H, S, R, and J), as well as with density and precipitation. This underscores that denser plants conditions and increased rainfall are conducive to the absence of dominant species and high diversity. The D index is correlated negatively with pH, T range, and loam.

The Margalef richness index (Figure 10c) shows a negative correlation with the range temperature, loam, OM-OC5, and C/N ratio, indicating that variations in carbon and nitrogen proportions influence species richness. Additionally, it is positively correlated with H, S, D, relative humidity, precipitation, clay, and density, suggesting that more humid conditions favor greater species richness.

The Shannon index (Figure 10c) shows a negative correlation with temperature range, C/N ratio, and the percentage of loam in the soil, indicating that extreme temperature fluctuations and loamy soils restrict species diversity. In contrast, it is positively associated with the percentage of clay, plant density, precipitation, relative humidity, and other diversity indices (D, R, and H). These associations illustrate that denser, clay-dominant soils, along with sufficient precipitation, promote higher biological diversity.

Finally, the specific richness index H (Figure 10c) is positively correlated with clay, density, precipitation, D, H, J, and R index. However, it is correlated negatively with loam, T rang, pH, and ratio C/N. These correlations indicate that denser conditions and high levels of precipitation not only promote diversity but also enhance the specific richness of ecosystems.

### 3.6. Differences in Community Composition

**Figure 11:**
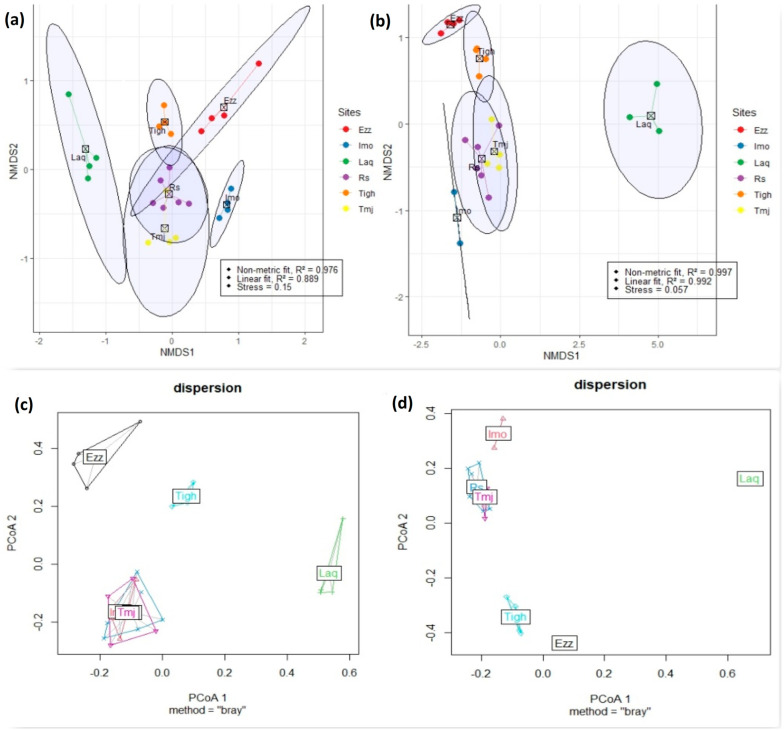
The biplot of the non-metric multidimensional scaling (NMDS) ordination and the biplot of the Dispersion ordination were prepared using the Bray–Curtis dissimilarity index to illustrate the variations in plant community composition across the sites in two years dependently, with the dashed lines representing the 95% confidence intervals. (**a**) NMDS plant community composition in 2021. (**b**) NMDS plant community composition in 2023. The stress value indicates the corresponding level of stress. (**c**) Dispersion plant community composition in 2021. (**d**) Dispersion plant community composition in 2023. Rs: Rasmouka, Tmj: Tamjloujt, Tigh: Tioughza, Laq: Laqsabi, Imo: Imoulass, Ezz: Ezzaouite.

The NMDS ordination revealed distinct patterns in species composition across the study sites. The analysis indicated that the year had a stronger influence on species composition at different sites. Stress values were observed to be 0.15 in 2021 and 0.05 in 2023 (K = 2) (Figure 11). These findings were further corroborated by PERMANOVA and ANOSIM analyses, which demonstrated a significant divergence in community structure between the sites over the two years. A *p*-value of <0.001 highlights a significant level of species heterogeneity among the sites (Table 7).

The NMDS analysis revealed varying degrees of overlap among the different sites in 2021, indicating compositional homogeneity across the studied sites (Figure 11a,b). In contrast, minimal overlap in total plant species composition was observed among sites in 2023, suggesting significant species heterogeneity at the site level. Additionally, the results of the PERMDISP analysis supported the overlap observed in the NMDS. While dispersion was not significant in 2021 (*p* = 0.554), it was significant in 2023 (*p* = 0.001). This suggests that dispersion did not influence compositional variations in 2021, but it did in 2023 (Figure 11c,d).

### 3.7. Comparison of Beta Diversity Indices in Different Sites for Each Year

**Figure 12:**
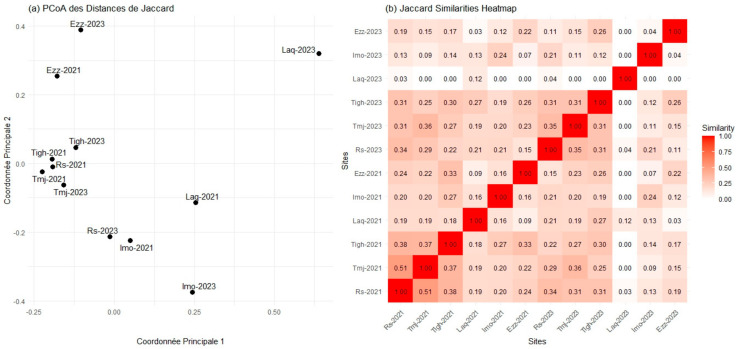
The graphs present the Jaccard similarity matrix.(**a**) The graph displays the results in two dimensions using principal coordinate analysis (PCoA), (**b**) The graph shows the results in a heatmap with values. Rs: Rasmouka, Tmj: Tamjloujt, Tigh: Tioughza, Laq: Laqsabi, Imo: Imoulass, Ezz: Ezzaouite.

A pairwise comparison of the differences between the floristic communities at the sites revealed a gradient of increasing dissimilarity, with greater differences observed between sites compared to within-site comparisons. The matrix, which displays the Jaccard distance values for the six sites over two years, provides a visual representation of these pairwise comparisons (Figure 12). It is important to note that the matrix is symmetrical, with values of 1 along the main diagonal, indicating self-comparisons where no differences are present.

The greatest degree of floristic overlap was observed between Rs-2021 and Tmj-2021, with a Jaccard distance value of 0.51. In contrast, the floristic composition differed the most between Laq-2023 and all other sites, except for Laq-2023, Rs-2023, and Rs-2021, with a Jaccard index value of 0.00 (Figure 12). The significant overlap in plant species composition between Rs-2021 and Tmj-2021 is likely due to their geographical proximity, similar rainfall, and shared seed stock, resulting in similar species present at both sites. The considerable difference in floristic composition between the Ezz and Laq plots can be attributed to the remnants of natural flora in Ezz, characterized by a larger number of species, as well as geographical, climatic, and soil differences between the sites.

The Jaccard distance metrics (Figure 12) are complemented by beta diversity, quantified using the Sorensen index (Figure S2), which displays the lower triangle of the pairwise comparison matrix. A value of 1 on the main diagonal indicates complete overlap in floristic composition within the same sites, while a value of 0 represents no overlap. The values in Figure S2 were further analyzed using a clustering algorithm and presented in a heatmap (Figure S2), offering a more graphical and visually appealing representation of the results. The outcomes of the Sorensen index are consistent with those of the Jaccard distance, showing the highest degree of overlap and distinction for the same pairs of plots. From a biodiversity perspective, areas with more diverse plant compositions are more beneficial than those with uniform plant components. This underscores the importance of incorporating beta diversity indices in habitat analyses.

In our study, Figure 11a–d clearly illustrate the differences in species distribution across the sites between 2021 and 2023, based on NMDS and dispersion results. In 2021, the sites, particularly Imo, Rs, and Tmj, are closely clustered, suggesting they share a higher number of common species. However, in 2023, a noticeable separation between the sites is observed, with Imo diverging significantly from Rs and Tmj. This shift reflects a decrease in shared species among the sites and the emergence of more distinct plant communities at each location.

The increase in disturbance at the ecosystem level appears to have driven this divergence. As disturbances intensify, plant diversity tends to decrease, favoring extreme selection processes that promote site-specific species colonization. This is evident in the growing differentiation between plant communities at each site, where fewer species are shared, and more specialized communities are established. The results highlight how disturbances can shape the composition and dynamics of plant communities over time, leading to more distinct ecosystems. Additionally, the results of the Jaccard and Sorensen similarity indices (Figure 12 and Figure S2) support these observations. These analyses, which focus on species presence and absence without considering abundance, show that in 2021, the Tigh and Tmj sites share a large number of common species, with Rs also exhibiting a similar pattern. However, by 2023, Rs shows a stronger similarity with Imo from 2021. The Laq site, in contrast, demonstrates very low similarity with Rs in both 2021 and 2023. Lastly, Ezz exhibits some similarity between the two years, though the number of species present has decreased in 2023. Together, these results highlight the changing species composition and the distinct ecological shifts occurring over time.

## 4. Discussion

In our study, we explored the dynamics of plant communities in argan tree (*Arganiaspinosa*) orchards between 2021 and 2023, as well as the impact of edaphic and climatic factors on biodiversity and their interactions. A total of 82 species from 25 families were identified, with 23 families observed in 2021 and 21 families in 2023. Notably, six of these species are endemic to Morocco: *Reseda diffusa*, *Chiliadenushesperius*, *Andryala integrifolia* subsp. *cedretorum*, *Rumexpapilio*, *Ononisnatrix* subsp. *arganietorum*, and *Frankenialaevis* subsp. *velutina*. Many of the plants in the study area are annual and biennial herbaceous species, and the plant communities show minimal seasonal stability. As precipitation decreased and temperatures increased, the biomass of annual and biennial herbs grew within the community, suggesting that these species have a competitive advantage in arid and warm environments [22]. Additionally, our study found that most of the species present are in high productivity stages.

Additionally, plants indirectly influence soils by depositing materials such as litter and root exudates on the soil surface, as well as through their nutrient absorption, all of which affect soil biogeochemical functions [23]. The argan region features diverse soils shaped by various physical factors, including relief, climate, and water balance. These variations create opportunities for different land uses [9]. The planting of argan trees has been integral in supporting this ecological transition. The argan tree is highly valued for its resilience and resistance to arid conditions [3]. Forest management, which includes various human activities and disturbances, plays a critical role in shaping the environmental factors that drive changes in plant diversity [23]. Furthermore, the argan ecosystem is essential in protecting soil from wind erosion and runoff while promoting groundwater recharge [9].

### 4.1. Relationships Between Plant Diversity, Density and Biomass Across Different Type’s Argan Orchards

Species diversity is a key index commonly used to assess sustainability across different ecosystem scales. It is a multifaceted parameter utilized to evaluate various plant-related indicators [24]. Our results showed a significant difference in the plant diversity among the argan orchards. However, the relationships between the Simpson index and biomass, the Shannon–Wiener index–biomass, and species richness–biomass exhibited negative correlation trends in 2021.

In this study, the evenness index showed no relationship with biomass across the various ecosystems in 2021 but was positively correlated in 2023. This aligns with the findings of Fayiah et al. [24], who stated that the relationship between plant diversity, evenness, and biomass can be either negatively linear or positively monotonic. Additionally, it was negatively correlated with density in 2021 and positively correlated in 2023. Although these species grow slowly and produce less biomass annually, they significantly contribute to the ecological diversity and long-term stability of the ecosystem. Various factors, including climatic conditions, soil characteristics, and biotic interactions, influence the seasonal dynamics in plant community structure and diversity [25]. Temperature and precipitation are the primary drivers of seasonal changes [26]. Environmental factors like soil properties also show considerable variation across the seasons [25]. Most studies have shown that plant diversity and density peak during the spring season [25], when favorable conditions support optimal growth [22]. The effects of climate on plant diversity patterns are typically explained by three main hypotheses: environmental energy, water–energy dynamics, and cold tolerance. In extremely arid regions, water is considered the primary limiting factor for plant diversity, strongly supporting the hydrothermal dynamic hypothesis [13]. Environmental hydrothermal conditions have important effects on the plant composition and productivity communities [22,27]. In Morocco, increasing rainfall variability has led to more frequent and prolonged droughts, complicating water management and the conservation of natural forests [10]. Currently, extensive research has been conducted on how environmental factors influence community stability. Communities characterized by high coverage, rich species diversity, adequate water and nutrient availability, and well-developed nutrient–energy cycling tend to exhibit strong resistance and resilience, leading to enhanced overall stability [28]. Numerous biodiversity experiments have demonstrated that higher species diversity enhances the stability of productivity over time, with the asynchronous responses of species to environmental fluctuations serving as a key underlying mechanism [29]. The Van der Plas [30], found that plant diversity exhibited a weak relationship with biomass production in natural plant communities, particularly in grasslands. It was found that plant diversity exhibited a weak relationship with plant biomass production in natural plant communities, particularly in grasslands.

In arid regions, community stability is influenced by various factors, including interspecies competition, environmental stress, and disruptive activities [28]. Interestingly, in these stressful environments, community stability is not necessarily linked to species richness or evenness [29]. Instead, research has shown that different species fulfill unique functions within ecosystems, which can be more critical to maintaining stability than species diversity alone. This emphasizes the importance of functional diversity, where the specific roles of individual species may have a greater impact on ecosystem resilience and diversity than the sheer number of species present [28]. Moreover, the development of species diversity in a plant community reflects a dynamic equilibrium process, where multiple species coexist through long-term interactions. These interactions involve differentiation and complementarity in the use of environmental resources, enabling species to share the same habitat. The distribution pattern of plants within the community is shaped by small-scale heterogeneity in habitat conditions, further contributing to the complexity of species coexistence and ecosystem stability [31].

Understanding how the relationship between elevation and alpha diversity (measured by the Shannon index) changes over time is crucial for anticipating future environmental impacts and adapting resource management strategies accordingly. In our study, however, we did not find a significant effect of elevation on the Shannon index, which contrasts with the findings of other research.

In many natural mountain habitats, alpha diversity generally follows a predictable trend: species diversity tends to be higher at mid-elevations than at lower altitudes. This increase in H′ at mid-altitudes is mainly attributed to favorable environmental conditions such as increased moisture availability and optimal temperatures that create an ideal habitat for a wide range of species to thrive and coexist [32].

Furthermore, environmental stressors can induce shifts in species distributions. For instance, when temperatures in grassland regions become excessively high, plant species tend to migrate to higher elevations. This migration leads to a decrease in species richness and alters the ecosystem’s species composition [33].

Additionally, while several studies have shown that most soil characteristics change with elevation—often following a clear upward or downward trend—the soil properties themselves do not exhibit a uniform pattern. Instead, they appear to be specific to particular altitudes. This variability is likely due to the complex interplay of multiple factors, including climate (e.g., temperature, precipitation), vegetation (e.g., plant habits, nutrient uptake strategies, secondary metabolites, litter decomposition patterns), and geology (e.g., soil fertility, mineral composition, rock formation, sediment deposition) [32].

In summary, although our results did not reveal a significant effect of elevation on alpha diversity, the general trends observed in natural mountain ecosystems—as well as the influence of environmental and soil factors—highlight the complexity of these systems. This underscores the importance of considering multiple interacting factors when assessing biodiversity patterns over time.

The variation in the plant diversity–biomass relationship in this study may be attributed to factors such as climate, geographic location, biogeographic history, and possibly grazing intensity.

### 4.2. Relationships Between Plant Productivity and Density

This study found a positive relationship between biomass and species density, a result that is consistent with and aligns closely with previous research findings [24]. Several studies have shown that the relationship between density and biomass varies significantly across different ecosystems [34]. Variations in plant density and fresh weight across different sites and years highlight the influence of local environmental conditions and soil properties. Consistent with Mi et al. [22], this study found that precipitation and temperature significantly influence the density and biomass of plant communities. Fresh weight was negatively influenced by maximum and mean temperatures, while precipitation showed no direct effect on biomass. These findings reinforce the idea that extreme temperatures limit plant growth, as evidenced in sites with higher temperatures. For instance, sites like Tigh, where plant density increased, continue to support the rapid growth of annual species, whereas sites like Laq, which experienced a significant decline in density, may be transitioning toward lower productivity stages. However, Desert soil is predominantly coarse, whereas meadow soil is loamy and rich in organic matter, which can affect the biomass [34]. Unlike previous findings and according to Fayiah et al. [24], who referenced other research, the lack of a relationship between biomass and plant cover, may be due to the influence of biotic and abiotic pressures.

Furthermore, the relationship between plant biomass and plant cover has been shown to depend on precipitation, temperature, topography, meteorological conditions, soil properties, and community dynamics. The arid and semi-arid argan biosphere is particularly sensitive to changes in temperature, water availability, and herbivore grazing over time.

### 4.3. Biomass, Diversity, Density, Soil Physiochemical Properties and Soil Texture

Our results highlighted differences not only in soil properties but also in structural features and plant diversity between our two years. In the present study, we found that out of six study sites, Imoulass and Ezzaouite had the higher plant biomass, which may be associated with the percentage of organic matter in the depth of 30 cm. The production of plant biomass plays a crucial role in carbon storage within terrestrial ecosystems, and its distribution is closely linked to the carbon cycle in grasslands [27]. Plant biomass is influenced by climatic conditions such as light, temperature, and water availability. The decrease in plant biomass could be attributed to climate change, and soil nutrient composition [24]. Correlation analyses support these findings. Plant density is positively correlated with soil clay content and, indicating that clay-rich soils and cooler temperatures promote higher plant density. Sites with high temperatures and sandy, bare soils create more fragile microclimatic conditions. As explained by Jentsch et al. [35], these conditions are marked by high surface temperatures (up to 70 °C in direct sunlight) and low water storage. The latter factor determines the uptake of nutrients by plants but can also lead to nutrient leaching from the soil, further reducing plant productivity.

Our results demonstrated that density and diversity indices (D, H, R, and S) were positively correlated with precipitation but negatively correlated with temperature range. In contrast, high temperatures seem to limit plant density, supporting the hypothesis that extreme temperatures reduce the competitiveness of species in arid environments. Fresh weight is positively correlated with elevation, relative humidity, and soil organic matter (OM-OC 30), suggesting that nutrient-rich soils and higher moisture levels promote biomass production. The relationship between plant diversity and biomass can be influenced by various environmental factors associated with changes in elevation within a diverse ecosystem [24]. Additionally, temperature decreases significantly with increasing altitude [36].

Soil develops through the combined effects of topography, climate, biological activity, parent material, and time, undergoing continuous changes in tandem with vegetative succession. Generally, positive succession enhances soil properties, while reverse succession leads to soil degradation [37]. Studies indicate that the connections between the diversity of plant species and soil nutrients are intricate and diverse. These interactions are complicated and rely on numerous factors. Soil supplies vital chemical elements and living conditions necessary for plant development, and soil nutrients play a crucial role in affecting plant growth within ecosystems. The presence of nutrients in the soil tends to have a positive correlation with plant growth [37]. Soil nutrient conditions can indicate the potential supply of essential elements. The carbon and nitrogen content in the soil may affect the composition of grassland plants and ecosystem functions [22]. As a key predictor and mediator of global changes affecting terrestrial ecosystems, shifts in soil pH can initiate alterations in biogeochemical cycles and biodiversity, which in turn can influence ecosystem biomass and its distribution [27]. Previous studies have shown that soil pH is the primary factor influencing vegetation richness [37]. Token et al. [28] demonstrated that soil phosphorus levels and pH directly affect plant distribution and community structure. Furthermore, soil heterogeneity in both nutrients and pH plays a crucial role in promoting plant coexistence and diversity. This has important implications for ecological restoration, where creating mosaic habitats can enhance species diversity [31].

No direct significant relationship between plant species diversity (H, S, D, and R) and soil nutrients was observed in this study, except for soil pH, which showed a negative correlation with biomass. Our findings indicate that the C/N ratio is negatively correlated with Margalef’s richness, particularly in environments with a high C/N ratio, which reduces nitrogen availability for plants. According to Liu et al. [27], soil fertility determines species diversity and productivity in grassland ecosystems, a result we confirmed in our study. Under conditions of low rainfall and a higher C/N ratio, the latter becomes a limiting factor for species richness. Furthermore, insufficient nitrogen restricts plant growth and biodiversity, leading to a decline in species richness [27]. However, these results should be further validated through additional research and ongoing studies to better understand the dynamics of the C/N ratio and its effects on biodiversity. The C/N ratio in soil has long been considered a key indicator of soil fertility and can be influenced by variations in soil pH [27].

Our study results indicate that soil pH and electrical conductivity (EC) have a direct influence on floral diversity in each area. Additionally, pH is affected by the increase in maximum temperatures. The availability of nutrients such as phosphorus, nitrogen, and organic carbon is the result of multiple ecological processes involving complex interactions among different soil organisms. However, the determinism of these soil-aggregated functions and the causal relationships between land-use practices, biodiversity processes, and ecosystem functions remain poorly understood [38]. The tools needed to understand biodiversity—its processes, functions, and services—must be applied locally, with a focus on soil processes, to provide a precise and comprehensive understanding of the causal relationships between local biodiversity and soil dynamics. Slight soil acidity plays a crucial role in this process. In soils with a pH below 7.20, levels of organic matter and organic carbon remain stable. A temporal evolution of organic matter has been observed in the first and second soil layers. Additionally, it has been shown that soil organic matter evolves in tandem with ecosystem maturation [38]. An increase in the C/N ratio at the site level within the same season across the two years has been shown to lead to a decrease in species diversity. This outcome can be attributed to the inhibitory effect of nitrogen uptake by plants [35]. According to Ma et al. [37], the correlation between nitrogen and phosphorus content was strong across the various soil layers. And this is confirmed in our study.

The high productivity of above-ground biomass is closely linked to soil stability. Accordingly, it can be posited that ecological rules impose an opposition between high productivity and the dynamic stability of natural ecosystems [38]. Plant biomass indicates the capacity of plants to absorb, accumulate, and utilize external nutrients for growth [28].

### 4.4. Impact of Climatic Complexity on Ecosystem Stability and Species Resilience in Arid Regions

Climatic complexity in vulnerable areas, such as arid regions, has a profound impact on ecosystem stability. Extreme conditions, including significant temperature fluctuations, limited water availability, prolonged droughts, and irregular rainfall, create a highly stressful environment for plant species, affecting both the stability and resilience of ecological communities. The climatic data for the sites studied in 2021 and 2023 show considerable variability in several key parameters. Maximum, minimum, and average temperatures differ significantly between sites, as does thermal amplitude, reflecting differences in microclimatic conditions. For instance, sites like Laqsabi, with higher maximum temperatures and lower relative humidity, experience increased heat stress. In contrast, sites such as Tioughza, benefit from more moderate climatic conditions.

These regional climatic variations highlight the heterogeneity of the environmental conditions to which plant communities must adapt. Species distribution is therefore closely linked to these climatic parameters, influencing the composition of communities and their ability to adapt to climatic and human disturbances. This heterogeneity partly explains why some species can persist in areas where climatic variability is more pronounced, while others are confined to more stable niches. Ultimately, these regional differences shape species adaptation and resilience, influencing the long-term stability of plant communities. Topography appears to emerge as a key determinant of ecosystem diversity, structure, and functioning [36].

## 5. Conclusions

In conclusion, biodiversity in argan orchards faces significant challenges due to topographic and climatic constraints, compounded by the impacts of human activities, resulting in severe biodiversity loss. This has led to a reduction in biodiversity, changes in ecosystem structure, and a weakening of ecosystem functions and services. Our study underscores the critical role desert plants play in maintaining biodiversity and ecological balance in arid environments. By examining the relationship between plant diversity and ecosystem function on a small scale within this vulnerable region, we have gained valuable insights into how these communities contribute to ecosystem stability and resilience. This research also provides a theoretical foundation for biodiversity management and conservation strategies.

The findings of our study highlight important shifts in plant community composition, with 82 species identified across 25 families, showing significant year-to-year fluctuations. The predominance of annual and biennial herbaceous species, which exhibit limited seasonal stability, reflects the influence of climatic variability, soil characteristics, and biotic interactions. Soil nutrients such as nitrogen, organic carbon, and phosphorus were positively correlated with plant biomass. Additionally, we observed that the relationships between plant density, biomass, diversity, and soil fertility varied significantly across orchard types at all six sites. The LMM results provide crucial information for understanding how environmental dynamics and other factors influence the density–FW relationship, with practical implications for resource management, policy and future research.

Future research should further investigate the influence of soil heterogeneity on plant communities across different spatial and temporal scales. It is also essential to explore the complex relationship between plant diversity and biomass, considering the varied environmental factors associated with elevation changes within diverse ecosystems. Continued conservation efforts are crucial to safeguarding the biodiversity and ecological integrity of the argan tree region, especially in the face of ongoing environmental challenges. These efforts are key to ensuring the sustainability of pastoral production and the long-term provision of vital ecosystem services.

## Figures and Tables

**Table 1:** Geographical locations and key characteristics of the six-argan orchards in the study area.

Site/Province	Code	Geographical Location	Elevation (m)	Soil Texture (%)			
				Sand	Loam	Clay	Class
Rasmouka/Tiznit	Rs	29°45′17.47″ N 9°32′1.96″ W	207	80.28	7.79	11.93	Loamy sand
Tamjloujt/Chtouka AitBaha	Tmj	29°57′53.34″ N 9°21′23.62″ W	239	57.61	29.53	12.86	Loamy sand
Tioughza/Sidi Ifni	Tigh	29°26′13.97″ N 10°1′55.73″ W	279	70.35	2.44	27.21	Loam clay sandy
Laqsabi/Guelmim	Laq	28°58′58.32″ N 10°15′31.33″ W	181	56.32	37.36	6.32	Loamy sand
Imoulass/Taroudant	Imo	30°45′33.1″ N 8°45′50.5″ W	1207	65.49	32.55	1.96	Loamy sand
Ezzaouite/Essaouira	Ezz	31°19′55.15″ N 9°30′20.47″ W	378	79.01	17.25	3.74	Sandy loam

**Table 2:** The T-test of the six sites physiochemical soil properties, between the years 2021 and 2023.

	Sites	Rs	Tmj	Tigh	Laq	Imo	Ezz
Soil properties	Pava (ppm)	(10.2880) ***	(0.2094) ^NS^	(9.9763) ***	(−1.5779) ^NS^	(2.2683) ^NS^	(−1.5541) ^NS^
	N %	(−0.4760) ^NS^	(−1.2445) ^NS^	(8.7930) ***	(0.9214) ^NS^	(−0.0334) ^NS^	(5.2588) **
	pH	(2.9085) *	(0.3801) ^NS^	(−0.6576) ^NS^	(−4.9930) **	(−0.4757) ^NS^	(0.3046) ^NS^
	EC (ds.m^−1^)	(−2.7005) *	(2.1488) ^NS^	(0.4460) ^NS^	(3.8122) **	(−1.1788) ^NS^	(−2.4613) *
	OM % (0–5 cm)	(−6.4614) ***	(−8.2672) ***	(−0.6603) ^NS^	(−0.7279) ^NS^	(0.2065) ^NS^	(−3.6207) *
	OM % (5–10 cm)	(−3.6687) *	(−9.2517) ***	(1.4533) ^NS^	(0.2566) ^NS^	(−1.7096) ^NS^	(−2.9597) *
	OM % (10–30 cm)	(−0.8909) ^NS^	(1.2525) ^NS^	(0.1886) ^NS^	(−1.0666) ^NS^	(−0.4379) ^NS^	(1.6333) ^NS^
	OC % (0–5 cm)	(−6.4612) ***	(−8.2664) ***	(−0.6599) ^NS^	(−0.7276) ^NS^	(0.2071) ^NS^	(−3.6198) *
	OC % (5–10 cm)	(−3.6685) *	(−9.2520) ***	(1.4537) ^NS^	(0.2568) ^NS^	(−1.7093) ^NS^	(−2.9593) *
	OC % (10–30 cm)	(−0.8902) ^NS^	(1.2530) ^NS^	(0.1889) ^NS^	(−1.0666) ^NS^	(−0.4378) ^NS^	(1.6340) ^NS^
	C/N %	(−2.3174) ^NS^	(−8.1231) ***	(−6.4848) ***	(1.1966) ^NS^	(−0.6605) ^NS^	(−6.6523) ***

(T value). (*** significant at *p* < 0.001; ** significant at *p* < 0.01; * significant at *p* < 0.05; NS: not significant *p* > 0.05). Rs: Rasmouka, Tmj: Tamjloujt, Tigh: Tioughza, Laq: Laqsabi, Imo: Imoulass, Ezz: Ezzaouite, OM: organic matter, OC: organic carbon, EC: electrical conductivity, Pava: available phosphorus.

**Table 3:** The linear mixed model examines the effect of plant density (Density) and year (Years) on fresh weight (FW) while accounting for variability between sites as a random effect.

	Fixed Factors		Random Factor
	Density	Years	Sites
Fresh Weight	t value = 3.100 *p* = 0.003	t value = −3.282 *p* = 0.002	Variance 0.7873

**Table 4:** The T-test of the six sites’ diversity indices between the years 2021 and 2023.

	Sites	Rs	Tmj	Tigh	Laq	Imo	Ezz
Diversity indices	Shannon Index	(2.867) *	(1.255) ^NS^	(1.257) ^NS^	(3.817) **	(1.338) ^NS^	(0.870) ^NS^
	Simpson Index	(2.376) *	(1.176) ^NS^	(1.079) ^NS^	(3.483) *	(1.067) ^NS^	(0.464) ^NS^
	Pielou Index	(0.970) ^NS^	(1.809) ^NS^	(−1.046) ^NS^	(1.107) ^NS^	(−1.194) ^NS^	(−0.548) ^NS^
	Margalef’s Richness	(3.682) **	(1.124) ^NS^	(2.020)^N S^	(2.081) *	(1.927) ^NS^	(1.391) ^NS^

(T value), (** significant at *p* < 0.01; * significant at *p* < 0.05; NS: not significant *p* > 0.05). Rs: Rasmouka, Tmj: Tamjloujt, Tigh: Tioughza, Laq: Laqsabi, Imo: Imoulass, Ezz: Ezzaouite.

**Table 5:** The linear mixed model showing the effect of fixed factors (Elv) and (years) on Shannon’s index of diversity (H′) while accounting for variability between sites as a random effect.

	Fixed		Random
	Elevation	Years	Sites
Shannon’s index of diversity (H′)	t value = 0.171 *p* = 0.873	t value = −4.347 *p* < 0.001	0.1133

**Table 6:** Mean monthly rainfall values (mm) for the six sites were recorded for each of the two years, 2021 and 2023.

	Ezz	Imo	Laq	Rs	Tigh	Tmj	*p*-Value
2021	46.01 ± 33.20 ^a^	38.01 ± 37.42 ^a^	16.0 ± 14.02 ^a^	22.01 ± 14.30 ^a^	27.01 ± 16.67 ^a^	46.40 ± 48.21 ^a^	0.502 ^NS^
2023	27.60 ± 42.03 ^a^	17.4 ± 28.88 ^a^	6.60 ± 8.47 ^a^	7.60 ± 8.91 ^a^	11.80 ± 16.83 ^a^	23.80 ± 34.07 ^a^	0.748 ^NS^

(^NS^: not significant *p* > 0.05). (^a^: results of test post hoc Duncan). Rs: Rasmouka, Tmj: Tamjloujt, Tigh: Tioughza, Laq: Laqsabi, Imo: Imoulass, Ezz: Ezzaouite.

**Table 7:** The results of the ANOSIM, PERMANOVA, and PERMDISP comparisons. The ANOSIM R-value indicates the differences between the groups, while the PERMANOVA F value represents the ratio of the differences between the groups to the differences within the groups. The R^2^ value indicates the ratio of between-group differences to total differences. The *p*-values for each pair indicate significance.

	ANOSIM		PERMANOVA			PERMDISP	
	R	*p*-Value	R^2^	F	*p*-Value	F	Pr(>F)
2021	0.918	0.001 ***	0.666	7.98	0.001 ***	0.831	0.554 ^NS^
2023	0.982	0.001 ***	0.802	16.25	0.001 ***	11.483	0.001 ***

(*** significant at *p* < 0.001; NS: not significant *p* > 0.05).

## Data Availability

All data generated in this work are provided within this manuscript.

## Supplementary Materials

The following supporting information can be downloaded at: https://www.mdpi.com/article/10.3390/plants14050664/s1, Figure S1: Total Rainfall (mm/season) in the studied sites. (Ezz: Ezzaouite, Imo: Imoulass, Laq: Laqsabi, Rs: Rasmouka, Tigh: Tioughza, Tmj: Tamjloujt). Figure S2: The two graphs present the Sorensen similarity matrix. The first graph displays the results in two dimensions using principal coordinate’s analysis (PCoA), while the second graph shows the results in a heatmap with values. Rs: Rasmouka, Tmj: Tamjloujt, Tigh: Tioughza, Laq: Laqsabi, Imo: Imoulass, Ezz: Ezzaouite.
