# Supplementary material for: Impact of Climate and Soil on Plant Dynamics and Ecosystem Stability in Argan Orchards
Source: Plants (Basel). 2025 Feb 21;14(5):664. doi: 10.3390/plants14050664 (PMC11902092; doi:10.3390/plants14050664)
Supplement: Supplementary file 1 [file plants-14-00664-s001.zip › plants-3385283-supplementary.pdf]

Supplementary Information:

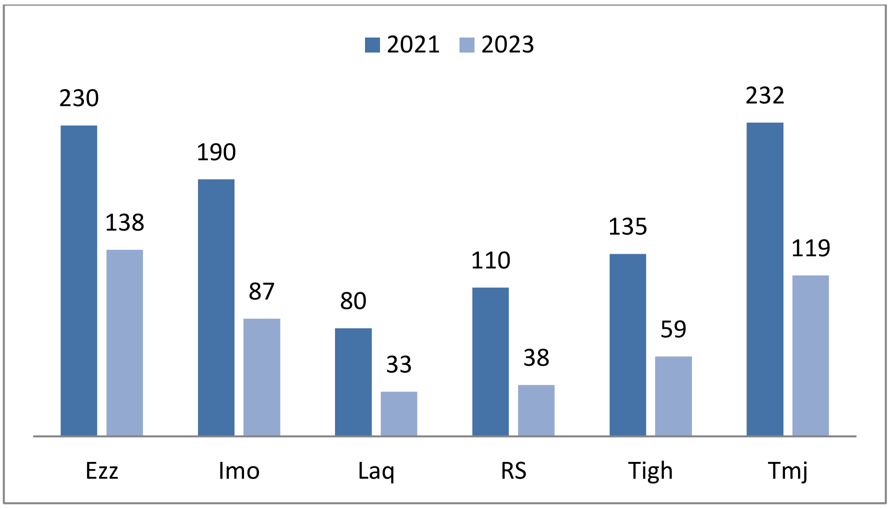

Figure S1. Total Rainfall (mm/season) in the studied sites. (**Ezz:** Ezzaouite, **Imo:** Imoulass, **Laq:** Laqsabi, **Rs:** Rasmouka, **Tigh:** Tioughza, **Tmj:** Tamjloujt).

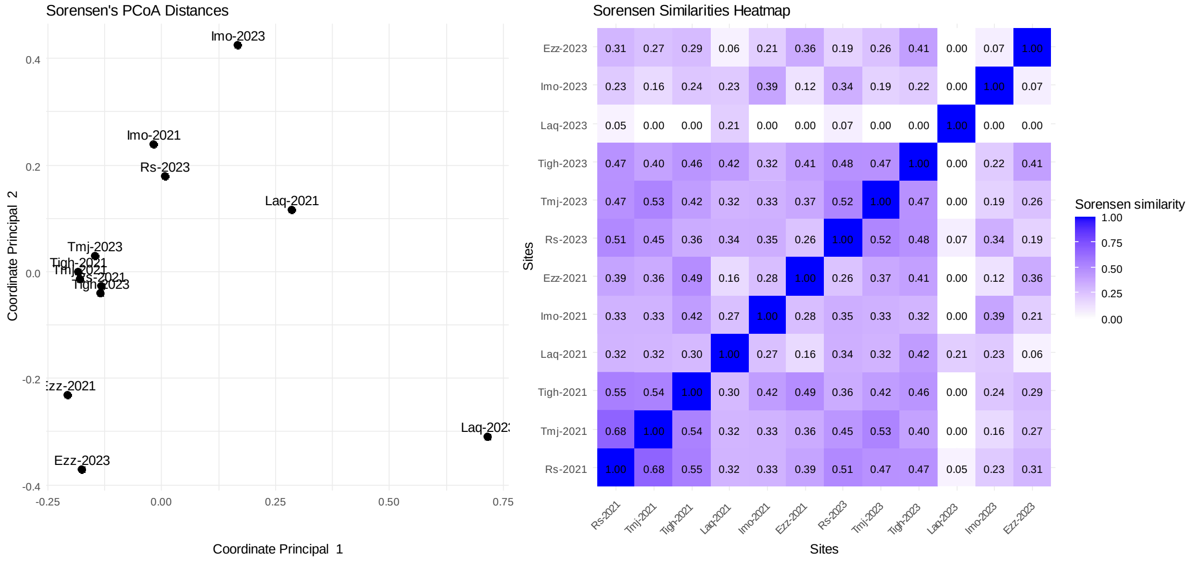

Figure S2. The two graphs present the Sorensen similarity matrix. The first graph displays the results in two dimensions using principal coordinate's analysis (PCoA), while the second graph shows the results in a heatmap with values. **Rs:** Rasmouka**, Tmj:** Tamjloujt**, Tigh:** Tioughza**, Laq:** Laqsabi**, Imo:** Imoulass**, Ezz:** Ezzaouite,
